# Acute Epiploic Appendagitis: A Nonsurgical Abdominal Pain

**DOI:** 10.1155/2019/7160247

**Published:** 2019-07-14

**Authors:** Marco Di Serafino, Francesca Iacobellis, Piero Trovato, Ciro Stavolo, Antonio Brillantino, Antonio Pinto, Luigia Romano

**Affiliations:** ^1^Department of General and Emergency Radiology, “Antonio Cardarelli” Hospital, Naples, Italy; ^2^Department of Advanced Biomedical Sciences, “Federico II” University Hospital, Naples, Italy; ^3^Department of Emergency Surgery, “Antonio Cardarelli” Hospital, Naples, Italy; ^4^Department of Radiology, Traumatology Centre “CTO-Dei Colli” Hospital, Naples, Italy

## Abstract

Epiploic appendagitis is a relatively rare disease characterized by an inflammation of fat-filled serosal outpouchings of the large intestine, called epiploic appendices. Diagnosis of epiploic appendagitis is made challenging by the lack of pathognomonic clinical features and should therefore be considered as a potential diagnosis by exclusion first of all with appendicitis or diverticulitis which are the most important causes of lower abdominal pain. Currently, with the increasing use of ultrasound and computed tomography in the evaluation of acute abdominal pain, epiploic appendagitis can be diagnosed by characteristic diagnostic imaging features. We present a case of epiploic appendagitis with objective of increasing knowledge of this disease and its diagnostic imaging findings, in order to reduce harmful and unnecessary surgical interventions.

## 1. Introduction

Epiploic appendagitis, also known as appendicitis epiploica, hemorrhagic epiploitis, epiplopericolitis, or appendagitis [1–3], is a relatively rare disease characterized by an inflammation of fat-filled serosal outpouchings of the large intestine, called epiploic appendices [2, 4, 5]. These adipose protrusions have normal length ranging from 5 mm to 5 cm and are distributed on the external surface of the cecum to the rectosigmoid in a number of 50-100 [6]. They are supplied by one or two arterioles and a single venule [7]. The appendagitis is caused by a spontaneous torsion causing obstruction of blood flow within the tissue and then ischemia up to a gangrenous necrosis of the appendage or by primary thrombosis of the draining vein and inflammation [6, 8]. The term “epiploic appendagitis” was introduced in 1956 by Lynn et al. [3, 9] and the computed tomography (CT) features were initially described in 1986 by Danielson et al. [10]. The most common sites of development of this disease are the rectosigmoid (57%) and the ileocecum (26%); rarer sites are the ascending (9%), transverse (6%), and descending colon (2%) [11–13]. Clinical presentation is typically characterized by acute or subacute abdominal pain, in most cases (60–80%) in the left lower quadrant, but it can also be localized in the right lower quadrant [14] miming a varied number of diseases such as appendicitis, diverticulitis, acute cholecystitis, and omental infarction [1, 13, 14]. Unlike its mimics, epiploic appendagitis is, generally, a self-limiting local inflammation and can be treated with anti-inflammatory medication [1, 15–20]. For these reasons, it is very important for clinicians to consider epiploic appendagitis as a cause for abdominal pain since a delay misdiagnosis can lead to prolonged hospital stay, antibiotic therapy, and surgical interventions [1, 12]. Today, ultrasound (US) and CT scan play a crucial role in diagnosis of this condition [16]. We present a case of epiploic appendagitis with objective of increasing knowledge of this disease and its US and CT findings, in order to reduce harmful and unnecessary surgical interventions.

## 2. Case Report

**Figure 1:**
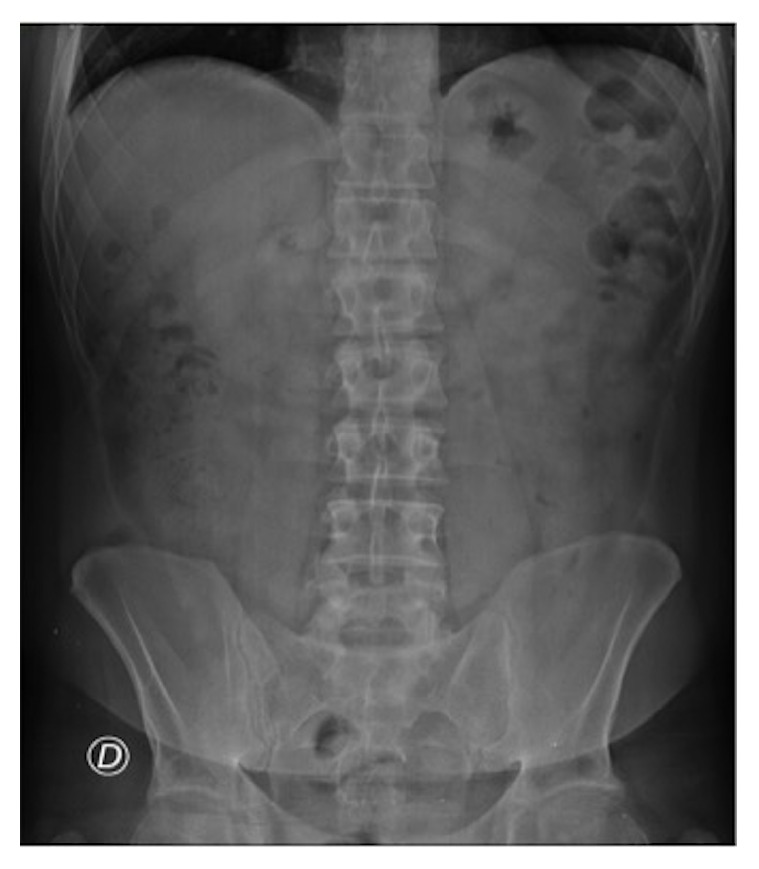
Spastic reflex ileum (gasless abdomen).

**Figure 2:**
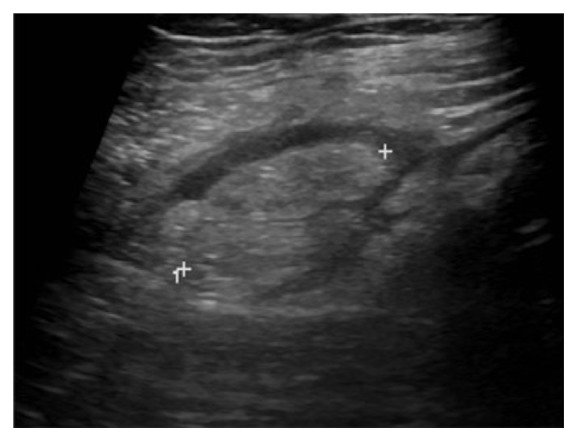
US image of the left lower quadrant with high frequency probe shows an oval noncompressible mass (calliper) with heterogeneous echotexture, located at the point of maximum tenderness.

**Figure 3:**
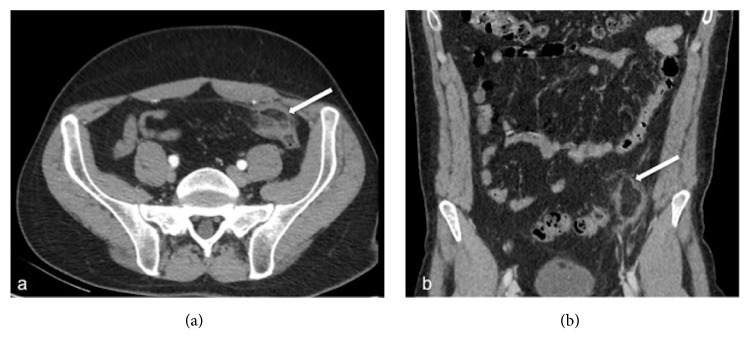
Axial (a) and coronal (b) contrast enhancement CT images show an oval pericolonic fat-density nodule (arrow) with a hyperdense ring and surrounding inflammation.

A 45-year-old Caucasian man presented to our emergency department (ED) with acute pain in the left iliac fossa that started the day before presentation. At clinical examination VAS (visual analogue scale) score was 7/10. He had fever and nausea and denied any associated chills, trauma to the area, vomiting, dysuria, haematuria, change in bowel habits, loss of weight, or skin rash. He also denied any history of renal colic. His family history was positive for gallbladder diseases requiring cholecystectomy; surgical history was negative and no chronic diseases were reported. At physical examination the patient showed tenderness and pain in the left iliac fossa associated with abdominal guarding, suggestive for diverticulitis. There was no pulsatile or palpable mass or costovertebral angle tenderness. Physical examination was otherwise unremarkable. The patient was placed on observation status and laboratory and diagnostic tests were ordered. The patient was treated with an intravenous (IV) bolus of 250 mL of normal saline solution followed by 125 mL/h IV normal saline solution and Ketorolac trometamina (Toradol® Roche Pharmaceuticals, Switzerland) 30 mg IV for pain control. Laboratory results showed White Blood Cell (WBC) count of 12,10 x 1000/*μ*l (4,8-10,8), with neutrophilia (87,3%) and fibrinogen of 839 mg/dL (160-350). Chest X-ray showed no lung consolidation, effusion, collapse, or air under the diaphragm. Abdominal X-ray was performed showing a poor representation of small and large bowel meteorism with no associated pathological air-fluid levels, as from spastic reflex ileus (Figure 1). An evaluation with abdominal US (Logiq e7™ GE Healthcare, USA) was performed using a high frequency linear probe (7,5 – 13Mhz) for the direct visualization of the descending and sigmoid colon in the left iliac fossa, because of the clinical suspicion of diverticulitis. US revealed a moderate reactive bowel wall thickening of the descending and the sigmoid colon with inflammatory change in the pericolonic fat, appearing as adjacent oval noncompressible hyperechoic mass, without internal vascularity and surrounded by a subtle hypoechoic line (Figure 2). According to the clinical conditions of the patient and the suggestive US findings, CT (128-slice Multidetector CT scanner GE Revolution GSI™, GE Healthcare, USA) scanning of the abdomen/pelvis with 120mL IV of iomeprol contrast media (Iomeron 400® Bracco, Italy) was also performed, confirming a moderate reactive wall thickening of the descending and the sigmoid colon with a nonenhancing adjacent fat-density ovoid structure characterized by a high-density rim and a surrounding inflammatory fat stranding (Figure 3). There was also CT evidence of colonic diverticulosis without CT evidence of diverticulitis. US and CT findings were most consistent with epiploic appendagitis. The patient remained under observation for 24 hours. Subsequently, upon symptoms improvement, the patient was discharged with a prescription for nonsteroidal anti-inflammatory medications and released into its family doctor's care.

## 3. Discussion

Epiploic appendagitis is a rare condition with an incidence of 8.8 per 1 million people and it is usually a diagnosis by exclusion [17]. Epiploic appendagitis may occur at any age. Two retrospective studies reported that men (70%) were affected more than women with an age range of 26 to 75 years [16]. As stated in a previous study by Son et al., there is no association with obesity [16, 18]. At clinical examination, patients usually describe a localized, strong, nonmigratory, sharp pain which usually started after a specific physical movement of their body, like postprandial exercise. An abdominal tenderness is present in all patients. There is a lack of fever, vomiting or leukocytic response [16]. Diagnosis of epiploic appendagitis is made challenging by the lack of pathognomonic clinical features and should therefore be considered as a potential diagnosis by exclusion. With diverticulitis and appendicitis being the most important causes of lower abdominal pain, they are the most frequent clinical diagnosis before diagnostic imaging or diagnostic laparoscopy. The pain usually is located on the left or right lower abdominal quadrant [16, 19] and, as reported in our case, the patient showed a very suggestive clinical finding for diverticulitis, with tenderness and pain in the left iliac fossa associated with abdominal guarding.

Currently, thanks to the increased use of US and CT in the evaluation of acute abdominal pain, most cases of epiploic appendagitis are diagnosed using CT (preferred) and US scan [20, 21]. Instead, Magnetic Resonance Imaging (MRI) is rarely used for diagnosis. Abdominopelvic US and CT examinations do not allow seeing the normal epiploic appendices, unless there is surrounding intraperitoneal fluid [22]. In cases of acute epiploic appendagitis, US evaluation shows, in the patient's area of maximal tenderness, the presence of a small (2-4 cm in maximal diameter) rounded or ovoid, noncompressible, and hyperechoic mass adherent to the colonic wall, without internal blood flow on color or power Doppler studies, frequently surrounded by a subtle hypoechoic line [11, 16, 22, 23]. The typical CT findings in cases of acute epiploic appendagitis include the presence of rounded or ovoid fat-density mass adjacent to the colonic wall, usually less than 5 cm in diameter (typical diameter range: 1.5–3.5 cm) [11, 19], the “hyperattenuating/hyperdense ring sign” [24], a hyperdense enhancing rim (thickness of 1-3 mm) surrounding the lesion, and the perilesional inflammatory fat stranding [11]. A pathognomonic CT finding of epiploic appendagitis is the “central dot sign”, characterized by a central, ill-defined round area of high attenuation within the fat-density mass [25, 26]. This sign is also known as the “dense central vessel sign” due to engorged or thrombosed vessel within the inflamed epiploic appendage [27]. Although the presence of this area of high attenuation is pathognomonic, its absence does not preclude a diagnosis of acute epiploic appendagitis [28].

MR may show a small oval mass with a signal intensity similar to the fat. Contrast-enhanced T1-weighted MRI images show also an enhancing rim around the oval fatty mass [1, 16]. 

The wall of the colon may show associated reactive thickening [11].

Chronically, a calcification can develop within the infarcted appendage epiploica and may detach to form an intraperitoneal loose body (peritoneal “mice”) [29]. Rarely, appendagitis may be located in the hernia sac [11] or involve the vermiform appendix, mimicking appendicitis [30].

Colonoscopy is sometimes performed before CT or US for the evaluation of abdominal colic pain; however, such a procedure will not provide an explanation for the presented symptoms in patients who have epiploic appendagitis [13]. In our case the US and CT findings were strongly indicative for appendagitis avoiding unnecessary invasive endoscopy.

Differential diagnosis for the imaging features of acute epiploic appendagitis include other acute inflammatory diseases, such as acute appendicitis, acute diverticulitis and sclerosing mesenteritis, fat-containing primary tumors or metastasis, and acute omental infarction, each one with characteristics imaging findings [11]. In particular, omental infarction is described as having many pathophysiologic similarities to epiploic appendagitis but, at CT, an omental infarction lesion is usually larger than that of epiploic appendagitis and is cake-like, centred in the omentum, and located medial to the cecum or ascending colon [13].

In the current literature, epiploic appendagitis is predominantly described as a self-limiting disorder and most patients are treated conservatively and nonsurgically either with or without nonsteroidal anti-inflammatory drugs as our case [13].

## 4. Conclusion

Unlike its mimics, such as appendicitis or diverticulitis, epiploic appendagitis is, generally, a self-limiting disease and is treated with anti-inflammatory therapy [1, 15, 16, 20]. Currently, with the increasing use of US and CT in the evaluation of acute abdominal pain, epiploic appendagitis can be diagnosed by characteristic diagnostic imaging features [11, 20]. For these reasons, the knowledge of epiploic appendagitis as a cause for abdominal pain and its imaging features may avoid a delay in diagnosis, unnecessary hospitalization, antibiotic therapy and surgical intervention [1, 14, 16].
